# Development and Validation of a Method for the Analysis of Bisoprolol and Atenolol in Human Bone

**DOI:** 10.3390/molecules24132400

**Published:** 2019-06-29

**Authors:** Lucia Fernandez-Lopez, Manuela Pellegrini, Maria Concetta Rotolo, Aurelio Luna, Maria Falcon, Rosanna Mancini

**Affiliations:** 1 Legal and Forensic Medicine, School of Medicine, University of Murcia,30100 Murcia, Spain; 2 National Centre on Drug Addiction and Doping, Istituto Superiore di Sanità, 00161 Rome, Italy

**Keywords:** beta-blockers, human bone, gas chromatography–mass spectrometry

## Abstract

A method based on gas chromatography–mass spectrometry (GC–MS) is described for the determination of bisoprolol and atenolol in human bone. After the addition of lobivolol as internal standard, pulverized samples were incubated in acetonitrile for 1 h under ultrasounds. After adjusting the pH of the samples to 6, they were centrifuged, and the supernatants were subjected to solid phase extraction. Elution was achieved by using 3 mL of 2% ammonium hydroxide in 80:20 dichloromethane:isopropanol solution. Eluted samples were evaporated and derivatized. Chromatography was performed on a fused silica capillary column and analytes were determined in the selected-ion-monitoring (SIM) mode. The assay was validated in the range 0.1–0.3 ng/mg (depending on the drug) to 150 ng/mg, the mean absolute recoveries were 60% for bisoprolol and 106% for atenolol, the matrix effect was 69% for bisoprolol and 70% for atenolol and process efficiency was 41% for bisoprolol and 80% for atenolol. The intra- and inter-assay accuracy values were always better than 12%. The validated method was then applied to bone samples from two real forensic cases in which toxicological analysis in blood were positive for atenolol in the first case (0.65 µg/mL) and bisoprolol in the second case (0.06 µg/mL). Atenolol was found in bone samples from the corresponding case at the approximate concentration of 148 ng/mg and bisoprolol was found at 8 ng/mg.

## 1. Introduction

In medico-legal death investigations, routine specimens typically collected at autopsies for toxicological analyses are blood, urine, gastric contents or vitreous humor. However, when long time has elapsed between death and sampling, these specimens are not available for the analysis due to the decomposition, so alternative matrixes are needed [1,2,3]. In case of significant putrefaction, skeletal tissues may provide the only source of information, since these specimens are well protected from decomposition [2,3,4,5]. In the last decade, skeletal tissues have been investigated as postmortem toxicological matrix by different authors who used a variety of methodologies for drugs extraction and detection [6,7,8,9,10,11,12,13,14,15,16]. Most researchers used experimental animals with the advantage of working under controlled conditions [2,4,6,7,8,9,10,11,12,13,14,15,16,17,18,19,20,21], while studies performed using human bones [1,5,22,23,24,25,26,27,28,29] are scarce and to date, do not use standardized protocols for sample preparation and analysis [2,28,29].

Hypertension is a major public health issue, since it already affects one billion people worldwide, leading to heart attacks and strokes. Raised blood pressure currently kills nine million people every year, being responsible for at least 45% of deaths due to heart disease and 51% of deaths due to stroke [30,31]. With an increasing number of people suffering from hypertension every year, the use of cardiovascular drugs such as beta-blockers has increased as well [32]. Beta-blockers have been widely used in the treatment of hypertension for more than three decades [33] and are still recommended by international guidelines [34,35]. Since their mechanism of action consists of blocking beta-adrenergic receptors, they are also indicated in the management of heart failure, coronary artery disease or cardiac arrhythmia as well to reduce cardiovascular complications in the perioperative period [36,37,38]. Beta-blockers are also used for psychiatric disorders such as anxiety, aggressiveness or tremor [39], and due to improved psychomotor performance these drugs are also on the list of substances prohibited in several sport competitions by the World Anti-Doping Agency, such as archery, golf or automobile [40]. 

Atenolol is one of the beta-blockers most used, it was among the 200 most prescribed drugs in the United States in 2003, reaching the fourth place in 2005, with 44 million prescriptions yearly [32,41,42]. However, while the consumption of atenolol has been progressively decreasing over the years, the consumption of bisoprolol has been increasing, being currently bisoprolol the most used antihypertensive drug in Spain followed by atenolol [43]. In spite of their security, they were among the substances associated with largest number of fatalities in 2017 [44] and different case reports of deaths where a beta-blocker is suspected to be the cause have been published [45,46,47,48,49]. 

In previous research in our laboratory bisoprolol and atenolol were detected in post-mortem blood from real forensic cases, so in this paper, a simple and rapid gas chromatography–mass spectrometry (GC–MS) method for the simultaneous analysis of bisoprolol and atenolol in human bone samples was developed. The GC–MS method was validated and applied to the real forensic cases whose toxicological analyses for these drugs in blood were positive.

## 2. Results

### 2.1. Gas Chromatography–Mass Spectrometry

**Figure 1:**
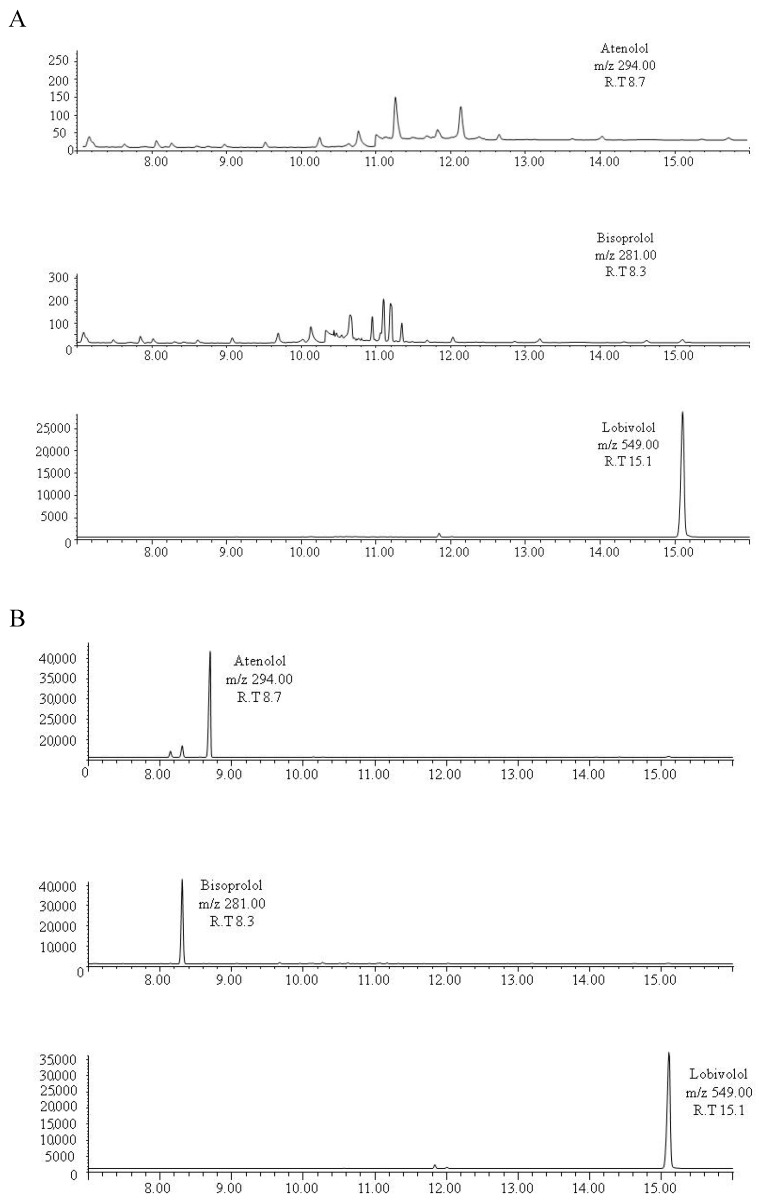
(**A**) Representative chromatograms obtained following the extraction of 300 mg of drug-free bone pool spiked with the internal standard lobivolol. (**B**) Selected ion monitoring chromatogram of an extract of 300 mg of drug-free bone pool spiked with 50 ng of bisoprolol, atenolol and lobivolol.

A chromatographic run was completed in 25 min. No additional peaks due to endogenous substances that could interfere with the detection of the substances of interest were found in drug-free samples. Representative chromatograms obtained following the extraction of drug-free bone pool samples are shown in Figure 1A. Possible interferences between the substances were not found as is shown in the selected ion monitoring (SIM) chromatograms of an extract of 300 mg of drug-free bone pool spiked with 50 ng of bisoprolol and atenolol (Figure 1B). Carryovers were not found in blank samples injected after the highest point of the calibration curve.

### 2.2. Validation Results

Validation data are summarized in Table 1 and Table 2. The determination coefficients (r^2^) of linear calibration curves for the substances under study in bone samples were equal to or higher than 0.990 up to 150 ng/mg bone samples. The limit of detection (LOD) and limit of quantification (LOQ) of each analyte were satisfactory for the purpose of the study (Table 1). The inter- and intra-assay accuracy values obtained for the quality control (QC) samples were in all cases better than 12% (Table 2). The mean absolute recoveries were 60% for bisoprolol and 106% for atenolol, the matrix effect was 69% for bisoprolol and 70% for atenolol and process efficiency was 41% for bisoprolol and 80% for atenolol. The differences in concentration of QC samples after any of the three freeze/thaw cycles were lower than 10% compared with the initial concentration. Results obtained in mid-term stability test were similar, with differences below 10%, ensuring the validity of the analysis of stored samples.

### 2.3. Application to Real Samples

**Figure 2:**
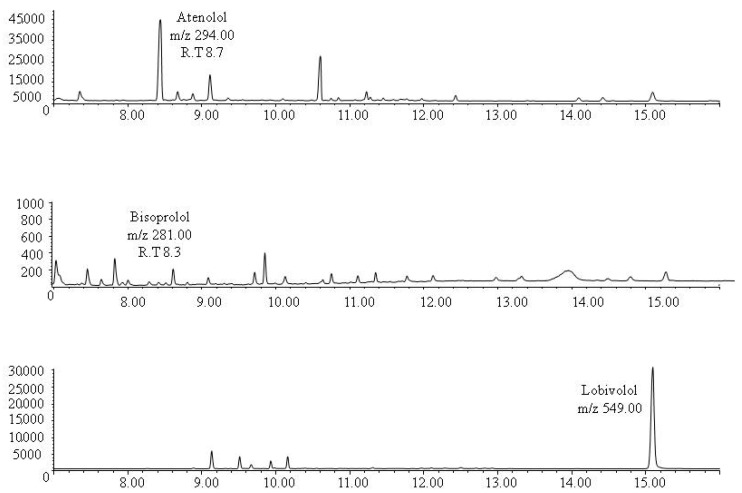
The SIM chromatogram of bones extracts from participant nº 1 containing approximately 148 ng/mg bone atenolol.

**Figure 3:**
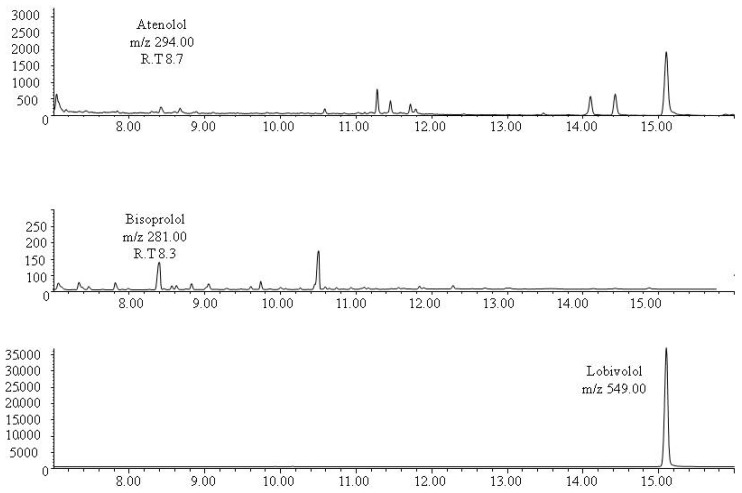
The SIM chromatogram of bones extracts from participant nº 2 containing approximately 8 ng/mg bone bisoprolol.

As the method showed satisfactory results in the validation protocol, it was applied to the analysis of real human bone samples taken from two corpses with positive blood toxicological tests for the drugs under study. The first case was positive to atenolol in blood at a concentration of 0.65 µg/mL which is within the therapeutic blood levels (0.1–1 µg/mL) and the second case was positive to bisoprolol with a blood concentration of 0.06 µg/mL which is also within the therapeutic blood levels (0.01–0.1 µg/mL) [50]. Both drugs were detected in the corresponding bone sample. Atenolol was found in bone at the approximate concentration of 148 ng/mg and the level of bisoprolol was 8 ng/mg. Representative chromatograms obtained following the extraction of real bone samples are shown in Figure 2 and Figure 3. 

## 3. Discussion

Atenolol and bisoprolol had been previously studied in postmortem specimens such as blood, urine or hair [32,40,48,51,52,53,54] but to our knowledge, they are analyzed in bone tissue here for the first time. At this time, these postmortem toxicological determinations in bone would inform about whether the deceased was exposed to the substance, but obtaining higher or lower levels would not report more information, since there is no standardized reference data of therapeutic and/or toxic bone concentrations of drugs in this matrix [50]. For this reason, the determination in future works of a correlation between drug concentrations in blood and bone would be very useful in these investigations, since it would give interpretative value to bone determinations. 

In this work, both drugs were found in bone in a lower concentration than in blood, similarly to previous cases analyzed in our laboratory with other substances [28,29]. This may be due to the short time between death and sampling in these cases, since there would not have been enough time for substances from the decomposition fluids to deposit in bone tissue and so beta-blockers would have reached the bone mainly by its own vascularization. In this way, the level of drugs in bone would depend on the corresponding level in blood, which in turn depends on the drug administration route, the dose, the patterns of consumption (acute vs. chronic), the time between last exposure and death, etc. [4,6,10,15,16,17]. Another factor to consider would be the extent of metabolism of each beta-blockers. While the less lipophilic beta-blockers such as atenolol are excreted unchanged in higher rates, the more lipophilic substances are extensively metabolized to produce more water-soluble derivatives, making more likely to detect the metabolites than the mother drug [55]. As we can see, many factors would influence in drug levels in bone making postmortem toxicological investigation in this matrix very complex and even more if we consider that the majority of these parameters (administration route, dose, patterns of consumption, time between last exposure and death) are usually unknown in forensic cases. 

Another consideration is the type of bone used for the analyses. The degree of vascularization of the bone depends on the anatomical site and the blood supply; moreover, trabecular bone is more vascularised than cortical bone due to the spongy structure [56]. Ribs are highly vascularised bones and contain considerable amount of bone marrow. A significant portion of the ribs is trabecular bone which allows a high contact surface of solid bone with bone marrow and blood and then with the eventual substances dissolved in them, making ribs a good option for toxicological investigations [29]. 

Cardiovascular drugs were among the substances most frequently involved in human poisonings in 2017 [44]. These poisoning have continuously increased since 2000, being beta-blockers among the substances associated with largest number of fatalities [44] and atenolol and bisoprolol among the beta-blockers most used. This work has demonstrated that atenolol and bisoprolol can be detected in bone tissue. This GC–MS method to analyse these beta-blockers in human bone was validated and tested in bone samples from forensic cases, evidencing the effectiveness of the method and the utility of human bone as toxicological matrix. However, further research is needed in this field increasing the number of patients and the beta-blockers analyzed.

## 4. Materials and Methods 

### 4.1. Chemicals and Reagents

Bisoprolol, atenolol and lobivolol (internal standard) were supplied by the Reina Sofia University Hospital (Murcia, Spain). All the reagents were of analytical grade and were obtained from Carlo Erba (Milan, Italy).

### 4.2. Bone Samples

The bone samples were obtained from corpses autopsied in the Legal Medicine Institute of Murcia, Spain. The sample consisted of ten corpses showing negative blood toxicological results to the drugs under study and two more corpses showing positive blood toxicological results to the drugs of interest. The majority of the deceased were male (75%); the average age was 55.9 ± 7.8 years (range 44–67 years) and the average time from death to sampling was 20.1 ± 7.8 h (range 2–32 h). Ethical approval was given by the Ethical Committee of the University of Murcia (ID: 1927/2018). 

The bone samples consisted of fragments of approximately 5 cm in length taken from central part of the fifth or sixth true rib. Samples from corpses with negative blood toxicological results were postulated as possible drug-free samples and, after verification, bone samples were mixed to obtain a homogeneous pool of blank samples to be used for calibration standards and QC. Samples from corpses with positive blood toxicological results were included in the study in order to apply the method to real forensic cases.

### 4.3. Preparation of Standard Solutions

Stock standard solutions of bisoprolol and atenolol were prepared in methanol at 1 mg/mL and maintained at −20 °C until analysis. Working solutions (100 and 10 µg/mL) were prepared by dilution with methanol. The IS lobivolol was prepared at a 50 ng/mL concentration.

Calibration standards were prepared daily for each analytical batch by adding to 300 mg of the pre-checked drug-free bone pool the suitable amounts of the methanol working solutions. The concentrations of the calibration standards were LOQ value, 10, 30, 50, 70 and 150 ng/mg bone. Three QC samples of each analyte were prepared at concentrations of 130 ng/mg (high quality control, QCH), 40 ng/mg (medium quality control, QCM) and 20 ng/mg (low quality control, QCL) in drug-free bone and stored at −20 °C.

### 4.4. Sample Preparation

Bone samples were conditioned as described in previous works [28,29]. Briefly, they were mechanically detached of remaining soft tissues and sectioned into small fragments, then they were dried at 50 °C for 24 h and pulverized using a ball mill (Millmix 20, Biogen, Madrid, Spain). Three hundred milligrams of the resulting bone powder with the IS working solution and 1.5 mL of acetonitrile were vortexed and incubated for 1 h under ultrasounds. Then, 2 mL of phosphate buffered saline (PBS; 0.1 M, pH 6) were added; samples were centrifuged at 3500 *g* for 5 min and the supernatants were recovered.

### 4.5. Solid-Phase Extraction

Samples were then applied to CleanScreen PKG50 extraction columns (3 cc, 200 mg, United Chemical Technologies, Bristol, PA, USA) for a solid phase extraction (SPE). Cartridges were sequentially preconditioned with 2 mL of methanol followed by 2 mL of PBS. Samples were loaded onto the cartridges and allowed to flow by gravity and the columns were then washed sequentially with 2 mL of distilled water and 1 mL of 0.1 M hydrochloric acid. Columns were dried under vacuum (10 in. Hg) for approximately 5 min and washed again with 1 mL of methanol. Elution was achieved by using 3 mL of 2% ammonium hydroxide in 80:20 dichloromethane: isopropanol solution. Eluents were evaporated to dryness at 40 °C under a gentle stream of nitrogen and 100 µL of *N,O*-bis(trimethylsilyl)trifluoroacetamide (BFTSA) 1% trimethylchlorosilane (TMCS) were added to dry extracts. Vials were then vortexed and derivatized for 30 min at 70 °C in capped test tubes. A 1-µL aliquot was injected into the GC–MS system.

### 4.6. Gas Chromatography–Mass Spectrometry Analysis

Analysis was carried out using a 6890 Series Plus gas chromatograph equipped with an Agilent 7683 autosampler and coupled to a 5973N mass selective detector (Agilent Technologies, Palo Alto, CA, USA). Data were analysed using the standard software supplied by the manufacturer (Agilent Chemstation).

Analytes separation was performed on a fused silica capillary column (ZB-SemiVolatiles, 30 m × 0.25 mm i.d., film thickness 0.25 μm, Phenomenex, Torrance, CA, USA). The oven temperature was programmed at 140 °C for 1 min, increased to 230 °C at 20 °C/min and held for 5 min, then raised to 290 °C at 20 °C/min and held for 20 min. Splitless injection mode was used. Helium (purity 99%), with a flow rate of 1 mL/min was used as carrier gas.

The temperatures of the injection port, ion source, quadrupole and interface were: 260, 230, 150 and 280 °C, respectively. The electron-impact (EI) mass spectra of the compounds were recorded in total ion monitoring mode (scan range 40–550 *m*/*z*) to determine retention times and characteristic mass fragments. Then, the instrument was operated in SIM mode. The qualifying ions monitored in SIM mode appear in Table 3; the underlined ions were used for quantification. The ion ratio acceptance criterion was a deviation of ≤20% of the average of ion ratios of all the calibrators.

### 4.7. Method Validation

The method was subjected to a validation protocol following the international criteria most recently reported in forensic toxicology [57,58]. Selectivity, carryover, linearity, LOD and LOQ, precision, accuracy, matrix effect, recovery, process efficiency and stability were determined as in previous works [28,29]. Validation parameters were calculated using five different daily replicates of the three QC samples over three subsequent working days. 

Selectivity was analysed by studying possible interferences due to endogenous substances and between the substances under study. Interferences due to endogenous compounds were assessed by measuring possible signals present at the retention times of the analytes under investigation and IS in the extracted pre-checked drug-free bone pool. By analysing the pre-checked drug-free bone pool with each compound added separately and all the compounds together, possible interferences between the substances were also determined. 

Calibration curves were performed in triplicate for all the substances and peak area ratios between the analytes and the IS were calculated. The linearity was admitted as acceptable when the coefficient of determination was above 0.990 and the calibrators were quantified within ±20% at the LOQ and ±15% at other concentrations. Five replicates of blank samples were analysed and standard deviation (S.D.) of the mean noise level at the retention time window of each analyte was used for determining LOD (LOD = 3 S.D.) and LOQ (LOQ = 10 S.D.). To investigate possible carryovers at the analytes retention times under investigation, the extracted drug-free bone pool only spiked with the IS was injected into the GC–MS system just after the analysis of the highest concentration point of the calibration curve. 

Precision and accuracy were analysed at the three QC concentrations and expressed as standard deviation and error (%) of the measured values, respectively. They were expected to be less than 20%. To determine the recovery, the matrix effects, and the process efficiency, the procedure proposed by Matuszewski et al. [59] was followed. In that protocol, three preparations were described: Set 1, Set 2 and Set 3. Set 1 was five replicates of QC material prepared in methanol and Sets 2 and 3 consisted of five replicates of the drug-free bone pool samples with QC material added after and before extraction, respectively. By comparing the mean peak area of analytes obtained in Set 3 (spiked before extraction) to those in Set 2 (spiked after extraction) recoveries were determined. By dividing the mean peak area of analytes obtained in Set 2 (spiked after extraction) to those in Set 1 (standard in methanol) matrix effects were calculated. And process efficiency was expressed as the ratio of the mean peak area of the analytes obtained in Set 3 (spiked before extraction) to those in Set 1 (standard in methanol). 

The stability of the analytes in bone after freeze (−20 °C) thaw cycles was evaluated by analysing of three cycles on QC samples. Moreover, mid-term stability was tested for real samples stored at −20 °C. Two samples were analysed in triplicate once a month over a period of six months. The stability was expressed as a percentage of the initial concentration (first analysed batch) of the analytes both in QC and real samples.

### 4.8. Expression of Analyte Levels

As described in previous studies using bone as toxicological matrix, the substance recovery from the bone tissue of corpses cannot be accurately determined due to the heterogeneity of the bone matrix, so drug levels in bone are reported as the response ratios (RR) normalized for the mass of tissue sampled (RR/m) [6,7,8]. The RR/m ratios are found proportional to the concentration in the validation protocol, so drugs levels can be compared with bone samples prepared in the same way over the range of concentrations assessed in the validation protocol. For better understanding, RR/m values have been expressed in concentration units (ng/mg), but they should only be used as approximations [7,12].

## Figures and Tables

**Table 1:** Linearity results, limits of detection (LOD) and limits of quantification (LOQ values for analytes under investigation.

Analyte	Calibration Parameters		LOD^b^ (ng/mg)	LOQ^b^ (ng/mg)
	Equation^a^	Determination Coefficients (r^2^) ^a^		
Atenolol	Y = 0.0368 x	0.997	0.1	0.1
Bisoprolol	Y = 0.0168 x	0.999	0.3	0.0

^a^ Mean of three replicates of calibration curves; ^b^ Mean of five replicates

**Table 2:** Intra- and inter-assay (*n* = 3) precision and accuracy obtained for analytes under investigation.

Analyte	Intra-Assay Precision (RSD)			Intra-Assay Accuracy (Absolute %Error)			Inter-Assay Precision (RSD)			Inter-Assay Accuracy (Absolute %Error)		
	QCL*	QCM**	QCH***	QCL	QCM	QCH	QCL	QCM	QCH	QCL	QCM	QCH
Atenolol	5	5.4	2.1	7.09	0.95	1.36	5.3	5.7	2.1	11.89	6.90	2.03
Bisoprolol	2.5	1.6	1.7	9.39	6.06	1.66	1.5	0.3	2.2	0.94	2.71	0.16

Relative standard deviation (RSD); *QCL: 20 ng/mg; **QCM: 40 ng/mg; ***QCH: 130 ng/mg.

**Table 3:** Retention times and characteristic ions of analyzed substances by gas chromatography–mass spectrometry GC–MS.

Substance	RT (min)	Characteristic Mass Fragments (*m/z*)
Atenolol	8.7	72-223-294-395
Bisoprolol	8.3	73–107–116-281
Lobivolol	15.1	73-294-549-534

RT: retention time. Underlined ions were used for quantification.
